# (*E*)-2-{(2-Hydroxy­naphthalen-1-yl)methyl­ene}hydrazinecarboxamide

**DOI:** 10.1107/S1600536809014561

**Published:** 2009-04-25

**Authors:** Yousef M. Hijji, Oyebola Oladeinde, Ray J. Butcher, Jerry P. Jasinski

**Affiliations:** a Department of Chemistry, Morgan State University, Baltimore, MD 21251, USA; b Department of Chemistry, Howard University, 525 College Street NW, Washington, DC 20059, USA; c Department of Chemistry, Keene State College, 229 Main Street, Keene, NH 03435-2001, USA

## Abstract

In the title mol­ecule, C_12_H_11_N_3_O_2_, the dihedral angle between the mean planes of the naphthalene and carboxamide groups is 28.9 (8)°. The hydrazine N atoms are twisted slightly out of the plane of the carboxamide group [C—C—N—N torsion angle = −175.06 (13)°]. The crystal packing is influenced by N—H⋯O hydrogen bonding which includes a bifurcated hydrogen bond between the amide N atom and nearby carboxyl and hydroxyl O atoms. A second bifurcated hydrogen bond occurs between the hydroxyl O atom and nearby amide (inter­molecular) and hydrazine (intra­molecular) N atoms. As a result, mol­ecules are linked into a co-operative hydrogen-bonded network of infinite one-dimensional O—H⋯O—H⋯O—H chains along the (101) plane of the unit cell in a zigzag pattern, the dihedral angle between the mean planes of the naphthalene groups of adjacent mol­ecules in the chain being 86.9 (2)°. A *MOPAC* PM3 calculation provides support to these observations.

## Related literature

For related semicarbazones, see: Noblia *et al.* (2005 ▶). For the bioactivity of semicarbazones, see: Beraldo & Gambino (2004 ▶). For their applications in polymers, see: Khuhawar *et al.* (2004 ▶) and in sensors, see: Oter *et al.* (2007 ▶).
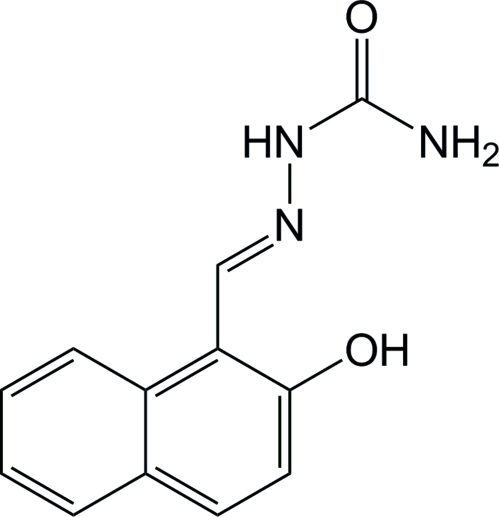


## Experimental

### 

#### Crystal data

C_12_H_11_N_3_O_2_
*M* *_r_* = 229.24
Monoclinic, 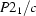

*a* = 16.0886 (4) Å
*b* = 4.72900 (10) Å
*c* = 15.6452 (4) Å
β = 114.647 (3)°
*V* = 1081.89 (5) Å^3^
*Z* = 4
Cu *K*α radiation
μ = 0.82 mm^−1^
*T* = 200 K
0.57 × 0.22 × 0.12 mm

#### Data collection

Oxford Diffraction Gemini R diffractometer
Absorption correction: multi-scan (*CrysAlis RED*; Oxford Diffraction, 2007 ▶) *T*_min_ = 0.819, *T*_max_ = 0.907
7383 measured reflections
2135 independent reflections
1724 reflections with *I* > 2σ(*I*)
*R*_int_ = 0.031

#### Refinement

*R*[*F* ^2^ > 2σ(*F* ^2^)] = 0.047
*wR*(*F* ^2^) = 0.124
*S* = 1.03
2135 reflections
155 parameters
H-atom parameters constrained
Δρ_max_ = 0.30 e Å^−3^
Δρ_min_ = −0.26 e Å^−3^

### 

Data collection: *CrysAlisPro* (Oxford Diffraction, 2007 ▶); cell refinement: *CrysAlisPro*; data reduction: *CrysAlis RED* (Oxford Diffraction, 2007 ▶); program(s) used to solve structure: *SHELXS97* (Sheldrick, 2008 ▶); program(s) used to refine structure: *SHELXL97* (Sheldrick, 2008 ▶); molecular graphics: *SHELXTL* (Sheldrick, 2008 ▶); software used to prepare material for publication: *SHELXTL* and *WebMOPro* (Schmidt & Polik, 2007 ▶).

## Supplementary Material

Crystal structure: contains datablocks global, I. DOI: 10.1107/S1600536809014561/rk2136sup1.cif

Structure factors: contains datablocks I. DOI: 10.1107/S1600536809014561/rk2136Isup2.hkl

Additional supplementary materials:  crystallographic information; 3D view; checkCIF report

## Figures and Tables

**Table 1:** Hydrogen-bond geometry (Å, °)

*D*—H⋯*A*	*D*—H	H⋯*A*	*D*⋯*A*	*D*—H⋯*A*
O1—H1*O*⋯N1	0.84	1.82	2.5562 (17)	146
N2—H2*A*⋯O2^i^	0.88	1.98	2.8290 (17)	161
N3—H3*A*⋯O1^ii^	0.88	2.10	2.9762 (18)	171
N3—H3*B*⋯O2^iii^	0.88	2.58	3.0618 (18)	116

Symmetry codes: (i) 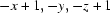; (ii) 
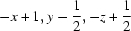
; (iii) 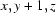.

**Fig. 1.**
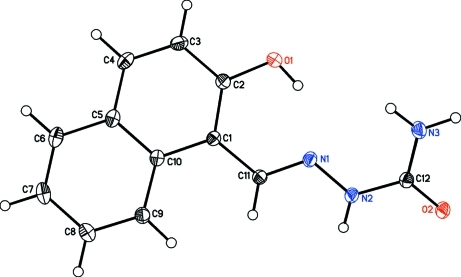
The molecular structure of C12H11N3O2, showing the atom numbering scheme. Displacement ellipsoids are drawn at 50% probability level. H atoms are presented as a small cyrcles of arbitrary radius.

**Fig. 2.**
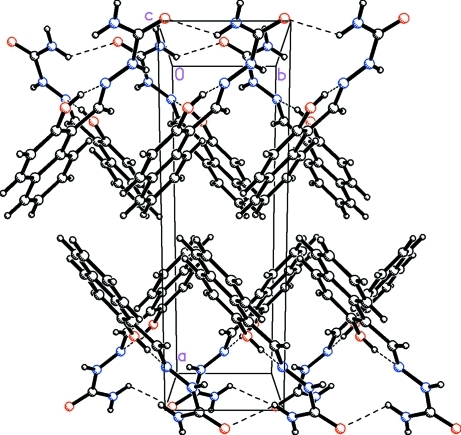
The molecular packing for C12H11N3O2 viewed down the c axis showing the cooperative hydrogen bond network of infinite one–dimensional O—H···O—H···O—H chains in a zigzag pattern. Dashed lines indicate intermolecular N—H···O and intramolecular O–H···N hydrogen bonds.

**Fig. 3.**
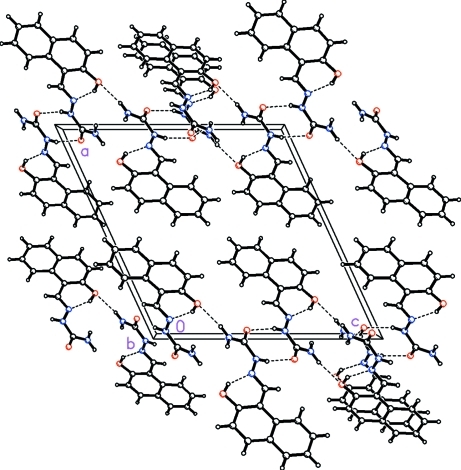
The molecular packing for C12H11N3O2 viewed down the b axis showing the cooperative hydrogen bond network of infinite one–dimensional O—H···O—H···O—H chains along the (1 0 1) plane of the unit cell in a zigzag pattern. Dashed lines indicate intermolecular N—H···O, and intramolecular O–H···N hydrogen bonds.

## supplementary crystallographic information

### Comment

The title compound, C_12_H_11_N_3_O_2_, is a tridentate semicarbazone ligand and forms complexes with a variety of metals. It coordinates with vanadium which forms complexes with potential antitumor activity (Noblia *et al.*, 2005). Semicarbazones show significant bioactivities including antiprotozoa and anticonvulsant types (Beraldo & Gambino, 2004), and additionally some derivatives have been used as selective fiber optic sensors for copper(II) (Oter *et al.*, 2007) or incorported into polymers (Khuhawar *et al.*, 2004).

The title molecule, C_12_H_11_N_3_O_2_, consists of a 2–hydroxynaphthalen–1–yl group and a hydrazinecarboxamide group bonded to a methylene carbon atom with the dihedral angle between the mean planes of the naphthalene and carboxamide groups measuring 28.9 (8)° (Fig. 1). The hydrazine nitrogen atoms are twisted slightly out of the plane of the carboxamide group [torsion angles C1–C11–N1–N2 = -175.06 (13)°]. The hydroxyl group is in the plane of the napthalene group [torsion angle = 179.62 (15)°]. Crystal packing is influenced by extensive strong intermolecular N—H···O hydrogen bonding which includes a bifurcated hydrogen bond between the amide nitrogen atom, N1, and a nearby carboxyl oxygen atom (O2) and hydroxyl oxygen atom (O1) (see Fig. 2, Table 1). A second bifurcated hydrogen bond occurs between the hydroxyl oxygen atom (O1) and nearby amide (N3) (intermolecular) and hydrazine (N1) (intramolecular) nitrogen atoms. As a result the molecules are linked into a cooperative hydrogen bond network of infinite one–dimensionsl O—H···O—H···O—H chains along the (1 0 1) plane of the unit cell in a zigzag pattern with the dihedral angle between the mean planes of the naphthalene groups of consecutive molecules in the chain measuring 86.9 (2)° (Fig. 3).

After a *MOPAC* PM3 calculation [Parameterized Model 3 approximation together with the Hartree–Fock closed–shell (restricted) wavefunction was used and minimizations were teminnated at an r.m.s. gradient of less than 0.01 kJ mol^-1^ Å^-1^] of the molecule in the asymmetric unit with *WebMO Pro* (Schmidt & Polik, 2007), the mean planes between the naphthalene and carboxamide groups changes from 28.9 (8)° to 14.8 (1)°, producing a significantly less twisted, more planar, molecule than that observed in the crystalline environment. It is apparent that the extensive hydrogen bonding scheme described significantly influences the crystal packing in the unit cell highlighted by a network of infinite one–dimensionsl O—H···O—H···O—H chains.

### Experimental

The title compound (I) was synthesized by adding a solution of 2–hydroxy–1–naphthaldehyde (1.72 g, 10 mmol) dissolved in 5 ml of ethanol to a solution of 1.15 g (10.4 mmol) of semicarbazide hydrochloride in 10 ml of water. The mixture was stirred at room temperature. A green precipitate formed. The mixture was stirred for 30 minutes then diluted with 50 ml of water, filtered and dried. Recrystallization from ethanol and slow evaporation of the solvent gave a light yellowish–green solid 1.55 g (68%). m.p. with decomposition > 503 K. ^1^H NMR (*DMSO*–d6, 400 MHz) δ (p.p.m.): 11.24 (br. s, 1H), 10.25 (br. s, 1H), 8.87 (s, 1H), 8.38 (d, J = 8.5 Hz, 1H), 7.85 (m, 2H), 7.55 (dt, J = 7.75, 1.4 Hz, 1 H), 7.37(t, J = 7.5 Hz, 1H), 7.17(d, J = 8.85, Hz, 1 H), 6.30 (br s, 2H); ^13^C NMR (*DMSO*–d6, 100 MHz) δ (p.p.m.): 156.07, 155.83, 139.86, 131.40, 131.31, 128.67, 127.94, 127.47, 123.26, 121.99, 118.44, 109.79.

### Refinement

The atoms H3A, H3B and H10 were obtained from a difference fourier map. The remaining H atoms were placed in their calculated positions and then refined using the riding model with C—H = 0.95 Å, and with *U*_iso_(H) = 1.18–1.21 *U*_eq_(C,N,O).

### Crystal data

**Table:**

C_12_H_11_N_3_O_2_	*F*(000) = 480
*M**_r_* = 229.24	*D*_x_ = 1.407 Mg m^−^^3^
Monoclinic, *P*2_1_/*c*	Cu *K*α radiation, λ = 1.54184 Å
Hall symbol: -P 2ybc	Cell parameters from 4156 reflections
*a* = 16.0886 (4) Å	θ = 5.2–73.5°
*b* = 4.7290 (1) Å	µ = 0.82 mm^−^^1^
*c* = 15.6452 (4) Å	*T* = 200 K
β = 114.647 (3)°	Needle, pale yellow
*V* = 1081.89 (5) Å^3^	0.57 × 0.22 × 0.12 mm
*Z* = 4	

### Data collection

**Table:**

Oxford Diffraction Gemini R diffractometer	2135 independent reflections
Radiation source: Fine–focus sealed tube	1724 reflections with *I* > 2σ(*I*)
Graphite	*R*_int_ = 0.031
Detector resolution: 10.5081 pixels mm^-1^	θ_max_ = 73.5°, θ_min_ = 5.7°
φ and ω scans	*h* = −18→20
Absorption correction: multi-scan (*CrysAlis RED*; Oxford Diffraction, 2007)	*k* = −4→5
*T*_min_ = 0.819, *T*_max_ = 0.907	*l* = −19→19
7383 measured reflections	

### Refinement

**Table:**

Refinement on *F*^2^	Primary atom site location: Direct
Least-squares matrix: Full	Secondary atom site location: Difmap
*R*[*F*^2^ > 2σ(*F*^2^)] = 0.047	Hydrogen site location: Geom
*wR*(*F*^2^) = 0.124	H-atom parameters constrained
*S* = 1.03	*w* = 1/[σ^2^(*F*_o_^2^) + (0.0706*P*)^2^ + 0.3033*P*] where *P* = (*F*_o_^2^ + 2*F*_c_^2^)/3
2135 reflections	(Δ/σ)_max_ < 0.001
155 parameters	Δρ_max_ = 0.30 e Å^−^^3^
0 restraints	Δρ_min_ = −0.26 e Å^−^^3^

### Special details

**Table:**

Geometry. All s.u.'s (except the s.u. in the dihedral angle between two l.s. planes) are estimated using the full covariance matrix. The cell s.u.'s are taken into account individually in the estimation of s.u.'s in distances, angles and torsion angles; correlations between s.u.'s in cell parameters are only used when they are defined by crystal symmetry. An approximate (isotropic) treatment of cell s.u.'s is used for estimating s.u.'s involving l.s. planes.
Refinement. Refinement of *F*^2^ against ALL reflections. The weighted *R*–factor *wR* and goodness of fit *S* are based on *F*^2^, conventional *R*–factors *R* are based on *F*, with *F* set to zero for negative *F*^2^. The threshold expression of *F*^2^ > σ(*F*^2^) is used only for calculating *R*–factors(gt) *etc*. and is not relevant to the choice of reflections for refinement. *R*–factors based on *F*^2^ are statistically about twice as large as those based on *F*, and *R*–factors based on ALL data will be even larger.

### Fractional atomic coordinates and isotropic or equivalent isotropic displacement parameters (Å^2^)

**Table:**

	*x*	*y*	*z*	*U*_iso_*/*U*_eq_	
O1	0.31323 (8)	0.7964 (3)	0.22177 (8)	0.0437 (3)	
H1O	0.3498	0.6774	0.2579	0.052*	
O2	0.56353 (8)	−0.0031 (2)	0.42849 (8)	0.0369 (3)	
N1	0.38963 (8)	0.4986 (3)	0.37070 (9)	0.0317 (3)	
N2	0.44479 (9)	0.2745 (3)	0.41411 (9)	0.0332 (3)	
H2A	0.4322	0.1678	0.4533	0.040*	
N3	0.54082 (10)	0.4022 (3)	0.34432 (10)	0.0391 (4)	
H3A	0.5881	0.3706	0.3312	0.047*	
H3B	0.5076	0.5557	0.3231	0.047*	
C1	0.26235 (9)	0.7936 (3)	0.34640 (10)	0.0280 (3)	
C2	0.25746 (10)	0.8928 (4)	0.26045 (10)	0.0325 (4)	
C3	0.19255 (11)	1.0963 (4)	0.20808 (11)	0.0391 (4)	
H3C	0.1902	1.1586	0.1494	0.047*	
C4	0.13328 (11)	1.2044 (4)	0.24071 (12)	0.0386 (4)	
H4A	0.0896	1.3413	0.2044	0.046*	
C5	0.13540 (10)	1.1162 (3)	0.32839 (11)	0.0325 (4)	
C6	0.07427 (11)	1.2315 (4)	0.36314 (12)	0.0408 (4)	
H6A	0.0312	1.3707	0.3274	0.049*	
C7	0.07638 (12)	1.1455 (4)	0.44726 (13)	0.0438 (4)	
H7A	0.0345	1.2225	0.4694	0.053*	
C8	0.14048 (12)	0.9437 (4)	0.50068 (12)	0.0414 (4)	
H8A	0.1419	0.8849	0.5593	0.050*	
C9	0.20126 (10)	0.8293 (4)	0.46977 (11)	0.0348 (4)	
H9A	0.2447	0.6942	0.5077	0.042*	
C10	0.20044 (9)	0.9091 (3)	0.38214 (10)	0.0287 (3)	
C11	0.32667 (10)	0.5704 (3)	0.39663 (10)	0.0291 (3)	
H11A	0.3220	0.4782	0.4484	0.035*	
C12	0.51931 (10)	0.2162 (3)	0.39678 (10)	0.0303 (3)	

### Atomic displacement parameters (Å^2^)

**Table:**

	*U*^11^	*U*^22^	*U*^33^	*U*^12^	*U*^13^	*U*^23^
O1	0.0468 (7)	0.0513 (8)	0.0418 (6)	0.0135 (6)	0.0272 (5)	0.0132 (5)
O2	0.0394 (6)	0.0298 (6)	0.0446 (6)	0.0091 (5)	0.0208 (5)	0.0026 (5)
N1	0.0305 (6)	0.0290 (7)	0.0354 (6)	0.0038 (5)	0.0135 (5)	0.0024 (5)
N2	0.0337 (7)	0.0283 (7)	0.0408 (7)	0.0063 (5)	0.0187 (6)	0.0074 (5)
N3	0.0418 (7)	0.0330 (8)	0.0523 (8)	0.0081 (6)	0.0293 (6)	0.0057 (6)
C1	0.0251 (7)	0.0271 (8)	0.0302 (7)	−0.0010 (6)	0.0098 (6)	0.0006 (6)
C2	0.0321 (8)	0.0330 (9)	0.0334 (7)	0.0003 (6)	0.0145 (6)	0.0011 (6)
C3	0.0410 (9)	0.0404 (10)	0.0333 (8)	0.0023 (7)	0.0129 (7)	0.0090 (7)
C4	0.0319 (8)	0.0347 (9)	0.0408 (8)	0.0056 (7)	0.0068 (6)	0.0065 (7)
C5	0.0252 (7)	0.0282 (8)	0.0400 (8)	−0.0020 (6)	0.0095 (6)	−0.0039 (6)
C6	0.0293 (8)	0.0355 (9)	0.0531 (10)	0.0042 (7)	0.0127 (7)	−0.0048 (7)
C7	0.0355 (8)	0.0447 (10)	0.0569 (10)	−0.0011 (7)	0.0249 (8)	−0.0148 (8)
C8	0.0410 (9)	0.0459 (10)	0.0426 (9)	−0.0037 (8)	0.0226 (7)	−0.0081 (8)
C9	0.0332 (8)	0.0363 (9)	0.0352 (8)	0.0024 (6)	0.0146 (6)	−0.0016 (6)
C10	0.0247 (7)	0.0263 (8)	0.0333 (7)	−0.0036 (6)	0.0103 (6)	−0.0045 (6)
C11	0.0293 (7)	0.0279 (8)	0.0296 (7)	−0.0002 (6)	0.0119 (6)	0.0002 (6)
C12	0.0305 (7)	0.0289 (8)	0.0310 (7)	0.0008 (6)	0.0125 (6)	−0.0044 (6)

### Geometric parameters (Å, °)

**Table:**

O1—C2	1.3534 (19)	C3—H3C	0.9500
O1—H1O	0.8400	C4—C5	1.421 (2)
O2—C12	1.2386 (18)	C4—H4A	0.9500
N1—C11	1.284 (2)	C5—C6	1.416 (2)
N1—N2	1.3674 (18)	C5—C10	1.424 (2)
N2—C12	1.3630 (19)	C6—C7	1.364 (3)
N2—H2A	0.8800	C6—H6A	0.9500
N3—C12	1.343 (2)	C7—C8	1.398 (3)
N3—H3A	0.8800	C7—H7A	0.9500
N3—H3B	0.8800	C8—C9	1.370 (2)
C1—C2	1.395 (2)	C8—H8A	0.9500
C1—C10	1.438 (2)	C9—C10	1.417 (2)
C1—C11	1.457 (2)	C9—H9A	0.9500
C2—C3	1.405 (2)	C11—H11A	0.9500
C3—C4	1.355 (2)		
			
C2—O1—H1O	109.5	C4—C5—C10	119.23 (14)
C11—N1—N2	118.82 (13)	C7—C6—C5	120.90 (16)
C12—N2—N1	120.02 (13)	C7—C6—H6A	119.6
C12—N2—H2A	120.0	C5—C6—H6A	119.6
N1—N2—H2A	120.0	C6—C7—C8	119.71 (16)
C12—N3—H3A	120.0	C6—C7—H7A	120.1
C12—N3—H3B	120.0	C8—C7—H7A	120.1
H3A—N3—H3B	120.0	C9—C8—C7	121.05 (16)
C2—C1—C10	118.52 (13)	C9—C8—H8A	119.5
C2—C1—C11	120.34 (14)	C7—C8—H8A	119.5
C10—C1—C11	121.10 (13)	C8—C9—C10	121.09 (15)
O1—C2—C1	122.44 (14)	C8—C9—H9A	119.5
O1—C2—C3	116.10 (14)	C10—C9—H9A	119.5
C1—C2—C3	121.45 (14)	C9—C10—C5	117.57 (14)
C4—C3—C2	120.48 (15)	C9—C10—C1	123.14 (14)
C4—C3—H3C	119.8	C5—C10—C1	119.29 (14)
C2—C3—H3C	119.8	N1—C11—C1	119.82 (13)
C3—C4—C5	121.03 (15)	N1—C11—H11A	120.1
C3—C4—H4A	119.5	C1—C11—H11A	120.1
C5—C4—H4A	119.5	O2—C12—N3	122.81 (14)
C6—C5—C4	121.10 (15)	O2—C12—N2	119.71 (14)
C6—C5—C10	119.67 (15)	N3—C12—N2	117.48 (14)
			
C11—N1—N2—C12	−171.55 (13)	C8—C9—C10—C5	1.2 (2)
C10—C1—C2—O1	179.81 (14)	C8—C9—C10—C1	−179.55 (15)
C11—C1—C2—O1	−2.4 (2)	C6—C5—C10—C9	−0.6 (2)
C10—C1—C2—C3	−1.4 (2)	C4—C5—C10—C9	179.18 (14)
C11—C1—C2—C3	176.35 (15)	C6—C5—C10—C1	−179.86 (14)
O1—C2—C3—C4	179.62 (15)	C4—C5—C10—C1	−0.1 (2)
C1—C2—C3—C4	0.8 (3)	C2—C1—C10—C9	−178.19 (14)
C2—C3—C4—C5	0.2 (3)	C11—C1—C10—C9	4.1 (2)
C3—C4—C5—C6	179.19 (15)	C2—C1—C10—C5	1.1 (2)
C3—C4—C5—C10	−0.5 (2)	C11—C1—C10—C5	−176.66 (13)
C4—C5—C6—C7	179.81 (15)	N2—N1—C11—C1	−175.06 (13)
C10—C5—C6—C7	−0.5 (2)	C2—C1—C11—N1	12.1 (2)
C5—C6—C7—C8	0.9 (3)	C10—C1—C11—N1	−170.23 (13)
C6—C7—C8—C9	−0.3 (3)	N1—N2—C12—O2	−172.55 (13)
C7—C8—C9—C10	−0.8 (3)	N1—N2—C12—N3	6.8 (2)

### Hydrogen-bond geometry (Å, °)

**Table:**

*D*—H···*A*	*D*—H	H···*A*	*D*···*A*	*D*—H···*A*
O1—H1O···N1	0.84	1.82	2.5562 (17)	146
N2—H2A···O2^i^	0.88	1.98	2.8290 (17)	161
N3—H3A···O1^ii^	0.88	2.10	2.9762 (18)	171
N3—H3B···O2^iii^	0.88	2.58	3.0618 (18)	116

Symmetry codes: (i) −*x*+1, −*y*, −*z*+1; (ii) −*x*+1, *y*−1/2, −*z*+1/2; (iii) *x*, *y*+1, *z*.
